# Changes in variance explained by top SNP windows over generations for three traits in broiler chicken

**DOI:** 10.3389/fgene.2014.00332

**Published:** 2014-10-01

**Authors:** Breno de Oliveira Fragomeni, Ignacy Misztal, Daniela Lino Lourenco, Ignacio Aguilar, Ronald Okimoto, William M. Muir

**Affiliations:** ^1^Department of Animal and Dairy Science, University of GeorgiaAthens, GA, USA; ^2^Instituto Nacional de Investigación AgropecuariaLas Brujas, Uruguay; ^3^Cobb Vantress Inc., Siloam SpringsAR, USA; ^4^Department of Animal Sciences, Purdue UniversityWest Lafaytee, IN, USA

**Keywords:** genomic selection, genome-wide association study, QTL, ssGBLUP, gene identification

## Abstract

The purpose of this study was to determine if the set of genomic regions inferred as accounting for the majority of genetic variation in quantitative traits remain stable over multiple generations of selection. The data set contained phenotypes for five generations of broiler chicken for body weight, breast meat, and leg score. The population consisted of 294,632 animals over five generations and also included genotypes of 41,036 single nucleotide polymorphism (SNP) for 4,866 animals, after quality control. The SNP effects were calculated by a GWAS type analysis using single step genomic BLUP approach for generations 1–3, 2–4, 3–5, and 1–5. Variances were calculated for windows of 20 SNP. The top ten windows for each trait that explained the largest fraction of the genetic variance across generations were examined. Across generations, the top 10 windows explained more than 0.5% but less than 1% of the total variance. Also, the pattern of the windows was not consistent across generations. The windows that explained the greatest variance changed greatly among the combinations of generations, with a few exceptions. In many cases, a window identified as top for one combination, explained less than 0.1% for the other combinations. We conclude that identification of top SNP windows for a population may have little predictive power for genetic selection in the following generations for the traits here evaluated.

## INTRODUCTION

Past studies of genomics in livestock usually focused either on best estimation of breeding values (Calus, 2010) or on identification of major single nucleotide polymorphism (SNP) (Goddard and Hayes, 2009). For the latter, the purpose is exploring associations between SNP and phenotypes to better understand the genetic architecture of a trait or to use identified major SNP for genetic selection. With important SNP identified, the selection can be performed with simple tests for a few SNP.

Genetic selection using major SNP is successful if they explain a sizeable portion of the genetic variation and if their effects change little over time. Earlier simulation studies showed that linkage disequilibrium (LD) identified in one generation decays very slowly over generations (Meuwissen et al., 2001; Solberg et al., 2009). However, under strong selection the decay is much faster (Muir, 2007). Therefore, newer studies advocate continuous genotyping and recalculation of SNP effects (Habier et al., 2007; Sonesson and Meuwissen, 2009; Wolc et al., 2011). While the selection pressure would act on the largest quantitative trait loci (QTLs), it is not clear how this would impact the identification and estimation of values for the top SNP that may indicate presence of QTLs.

Identification of an individual SNP linked to a QTL is difficult because of the high collinearity of SNPs. SNPs may be in LD with a QTL so windows of consecutive SNPs can capture the effect of a QTL better than a single SNP (Habier et al., 2011). Also, SNP segments are useful to discriminate important effects from statistical noise (Sun et al., 2011). Bolormaa et al. (2010) looked at SNPs within 1 Mbp intervals. Peters et al. (2012) used windows of five adjacent SNP. In a simulation study, effects of individual QTL were best explained by the combined effect of eight adjacent SNP (Wang et al., 2012). The optimal window size may also be a function of effective population size (Goddard, 2009).

There is a shortage in studies searching for stability of marker effects across generations in production traits for broiler chicken. Despite this, in a layer population, Wolc et al. (2012) found that 1 Mbp SNP windows with large effects had consistent effects across generations, but windows that explained little variance of the trait were not validated. If a window effect is constant across generations or subsets of population, it can be indicative of a causative gene on that trait; however, if the effect is not robust, it can correspond to an unstable, sample-specific association that is not expected to provide good out-of-sample predictions.

One common issue on genome association studies is the large number of false positive gene discovery. Information from the chicken QTL database (Hu et al., 2013) shows a large number of QTL described—2,467 for growth traits, 68 for meat quality traits, and 28 for conformation—but few of these have been validated or reproduced by other studies. This can be observed not only in chicken, but in studies on all livestock species. In this way, GWAS results should be carefully interpreted before considering an association as a causative effect. A possible causative effect should be easily accessed in further assays considering similar population structure.

The purpose of this study was to identify SNP windows that explain major portions of genetic variance and see if those values are preserved during a course of selection for growth in chicken.

## MATERIALS AND METHODS

The data was provided by Cobb-Vantress Inc. (Siloam Springs, AR, USA). A total of 294,632 phenotypes from a pure line of broiler chicken collected across five consecutive generations (G1, G2, G3, G4, and G5) were used in this study. This was the sire line, selected mainly for growth rate, meat yield, feed conversion and livability, and secondarily for reproduction traits. The numerator relationship matrix included 297,017 animals. For the first two generations, animals were selected for genotyping based on body weight and conformation scores; leg defects were very unlikely. The remaining animals (from G3 to G5) were randomly selected for genotyping. The number of animals in each generation are shown in **Table 1**. The number of observations, means, and SD for all the traits are shown in **Table 2**.

**Table 1:** Number of animals with phenotypes and genotypes in each generation.

Generation	Phenotypes	Genotypes
G1	95,770	1,142
G2	72,795	1,165
G3	66,241	754
G4	52,808	801
G5	7,018	1,004
Total	294,632	4,866

**Table 2:** Number of observations, mean, and SD for the three traits.

Trait	Observations	Mean	SD
Body weight	294,632	92.66	17.2
Breast meat	75,377	45.68	7.22
Leg score	294,632	1.17	0.38

Initially, genotype information from 4,922 animals in a chip with 57,635 SNPs was available (Groenen et al., 2011). The genomic data was subject to a quality control (QC) before the analysis. This QC removed SNPs with minor allele frequency <0.05, with call rates <0.9, and monomorphic SNPs. It also removed genotypes with call rates <0.9. After QC, the genotype file had 4,866 animals genotyped for 41,036 SNPs.

SNP solutions were estimated by ssGWAS (genome-wide association study using a single-step BLUP approach; Wang et al., 2012; Dikmen et al., 2013). In this methodology, the data was initially analyzed by a multi-trait single-step genomic BLUP (ssGBLUP; Misztal et al., 2009; Aguilar et al., 2010) with the same model as used for BLUP analyses (Chen et al., 2011). Effects in the model included sex, contemporary group, animal additive, and maternal permanent environmental effects. Concerning the genomic information, the genomic relationship matrix (G) was scaled for the average of the numerator relationship matrix for the genotyped animals (**A_22_**), which took into account the effect of non-random genotyping caused by selection (Vitezica et al., 2011). Subsequently, EBV for genotyped animals (GEBV) were converted to SNP effects and weights of SNP effect were refined iteratively. The procedure followed the S1 scenario described in Wang et al. (2012), with GEBV computed once and SNP weights refined through three iterations. The equation for predicting SNP effects using weighted genomic relationship matrix was (Wang et al., 2012):

$$\overset{\wedge }{u}=D\,Z\prime \,{\left[Z\,D\,Z\prime \right]}^{-1}\,\overset{\wedge }{a_{g}}$$

In which: $\overset{\wedge }{u}$ is the vector with estimated SNP marker effects, D is a diagonal matrix of weights for variances of SNP effects, Z is a matrix relating genotypes of each locus to each individual, and ${\overset{\wedge }{a}}_{g}$ is the additive genetic effect for genotyped animals.

The individual variance of SNP effect (the same as in D) was estimated as (Zhang et al., 2010):

$${\overset{\wedge }{\sigma }}_{u,i}^{2}={\overset{\wedge }{u}}_{i}^{2}\,2\,p_{i}\,\left(1-p_{i}\right)$$

In which: ${\overset{\wedge }{u}}_{i}^{2}$ is the square of the *i*th SNP marker effect, *p*_i_ is the observed allele frequency for the second allele of the *i*th marker in the current population.

When windows of *n* adjacent SNPs were used; the variances attributed to them were calculated by summing the variance of the next *n* SNPs, for each SNP. Next, the combination that contained the highest values for exclusive windows was chosen to avoid double counting. It could happen that some windows had less than *n* SNPs if they were between two windows explaining more variance or in a window at the end or beginning of a chromosome. However, those smaller windows do not explain significant part of the variance.

The analyses were performed in four scenarios: complete data set; only genotypes and phenotypes from generations G1, G2, and G3; generations G2, G3, and G4; and from generations G3, G4, and G5. Numerator relationship matrix was complete in all scenarios. All ssGWAS computations were performed using the BLUPF90 family programs (Misztal et al., 2002) modified to account for genomic information (Aguilar et al., 2010).

The choice for ssGWAS was due to its ability to support phenotypes from ungenotyped animals directly, to handle multiple trait models, and to avoid spurious solutions on SNP effects due to sampling. Sampling in Bayesian alphabet family models is strongly dependent on priors and may produce spurious SNP estimates (Gianola et al., 2009; van Hulzen et al., 2012). Comparing GWAS models in a simulated population, Wang et al. (2012) showed that ssGWAS was the most accurate method to capture the effect of potential QTLs; windows of SNP effects were used in their study.

## RESULTS

**FIGURE 1:**
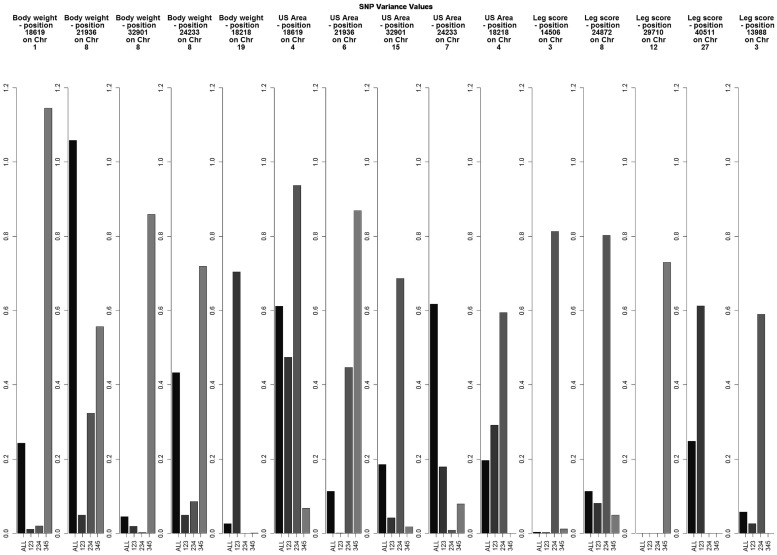
**Variance explained by the top five individual SNPs based on the combined results for all data sets for each trait**.

Preliminary results showed small individual SNP variances for all three traits, with just a few SNPs explaining more than 0.5% of the variance of the trait (**Figure 1**). Experiments with different SNP window sizes exhibited large noise with small sizes and absence of peaks with large sizes. Subsequently, windows of 20 SNP were chosen as a reasonable size.

**FIGURE 2:**
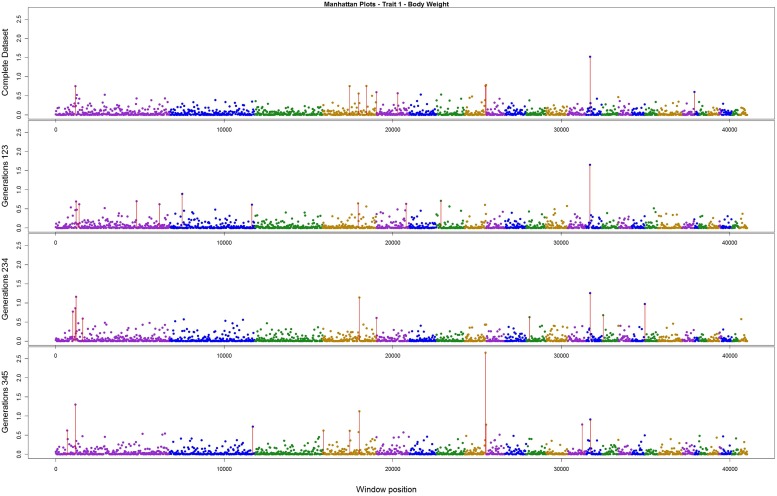
**Manhattan plots for percentage of variance explained for Body weight, performed for all the data set, and the subsets of generations**.

**FIGURE 3:**
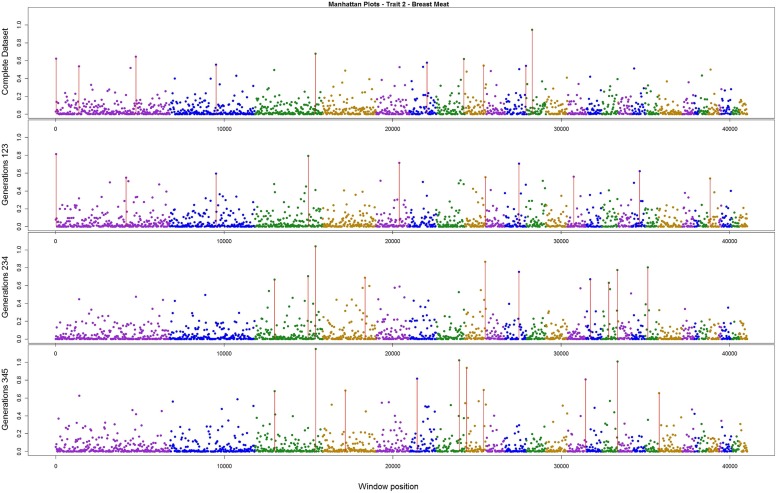
**Manhattan plots for percentage of variance explained for Breast meat, performed for all the data set, and the subsets of generations**.

**FIGURE 4:**
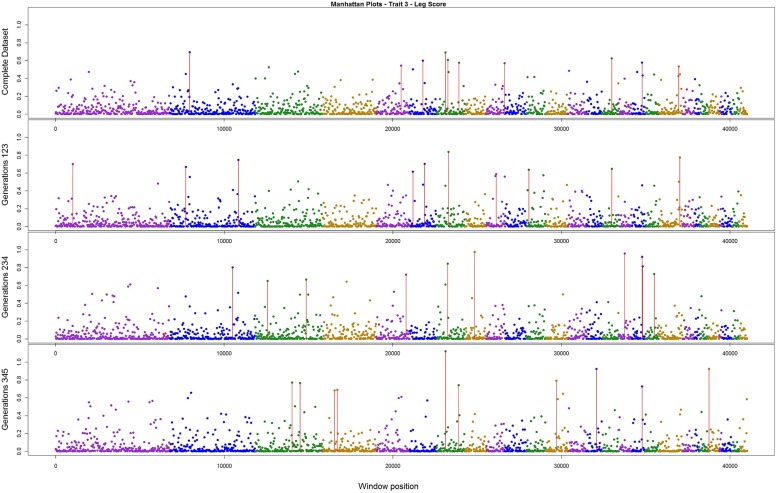
**Manhattan plots for percentage of variance explained for Leg score, performed for all the data set, and the subsets of generations**.

The variance explained by each SNP window is shown in **Figures 2–4** (corresponding to body weight, breast meat, and leg score, respectively); also, the 10 largest points were marked with a red vertical line. It is possible to see that all those traits are mainly affected by many regions with small effects, with few regions that explain more variance. These regions tended to change across the generations, but some of them retain a consistent value among the top 10 regions in all the scenarios, even though, the variance explained by those windows did not contribute significantly to the genetic variability of the trait.

For body weight, there were three regions that persisted among the top 10 in all the scenarios (**Figure 2**). Although these top three regions have been described before, the percentage of variance explained was small; only one region was above 2.5% and all the others were below 1.6%. The total variance explained by the top 10 windows summed up to 7.63%.

For breast meat, two regions were consistent among the scenarios (**Figure 3**). The window with larger effect for this trait explained 1.14% of the total variance, in the subset containing generations 3–5. The other windows explained at most 1%. The total variance explained by the top 10 windows was 6.26%.

For leg score, the value of just one region was constant across the analysis in chromosome 7 (**Figure 4**), the variance explained by this windows was 1.12% in the subset containing generations 3–5. All the other windows explained less than 1% of the genetic variance for this trait. The total variance explained by the sum of the top 10 windows was 6.01%.

## DISCUSSION

In our study, the three persistent regions observed for body weight could be related with QTLs previously described in the literature. The region in chromosome 1 was consistent with the one described by Carlborg et al. (2003) that associated this with a QTL responsible for body weight. The region in chromosome 4 can be related with those found by Carlborg et al. (2004), Ikeobi et al. (2004), and Ankra-Badu et al. (2010), all of whom detected a QTL for body weight in this region. The region in chromosome 14 was close to that described by Jennen et al. (2004) and Carlborg et al. (2003) for body weight. For breast meat, the region in chromosome 3 was close to those reported by Ikeobi et al. (2004) and Uemoto et al. (2009) for pectoralis muscle mass, and to those found by Gao et al. (2011) for chest width. The other region, in chromosome 8, was related by Ikeobi et al. (2004) to the pectoralis muscle mass trait. For leg score, the region in chromosome 7 had no relationship with any QTLs described previously in the literature for this trait in chicken. Nevertheless, there is a sequence of homeobox genes in the region around 16 Mbp in the same chromosome in the chicken genome. These homeobox genes (HOXD4, HOXD8, HOXD9, HOXD11, HOXD12, and HOXD13) are related with regulation of anatomical development, and might have a relationship with the leg disease score (Hillier et al., 2004). Thus, the findings in the current research are in concordance with Hayes and Goddard (2010), that a small number of markers with validated associations would explain a small portion of the genetic variance in the trait.

Wolc et al. (2012) found that for egg traits in layer chicken most of the SNPs with large effect were consistent across six generations, in both training and validating datasets. These findings could not be supported by the present results. Even though variances from three windows for body weight, two for breast meat, and one for leg disease score in the present study were stable across generations, for the other regions the results were different; it is possible that the lack of regions with larger effect on these traits, as illustrated in **Figures 2–4**, is the reason for the difference in findings. Another possible reason is the method used by the aforementioned authors; they used the Bayes B method, which assumes large effect for a few markers and is highly influenced by the prior information (Gianola et al., 2009; van Hulzen et al., 2012). In addition, the generation interval in layer chicken is a few times longer than in broiler chicken so their generations may have been overlapping. Yet, the genetic architecture could be different among the traits in the present study and in the aforementioned work.

Large changes in the variance explained by SNP windows could be indirectly due to small effective population size and subsequent low number of independent chromosome segments. According to Goddard (2009) and supported by Daetwyler et al. (2010), the number of such segments (*q*) is equal to 2N_e_L/log(4N_e_L), where *N_e_* is the effective population size and *L* is the length of chromosome in Morgans. Assuming *N_e_* = 50 (lower range showed in Andreescu et al., 2007) and *L*= 39, *q* = 435. Subsequently there are >100 SNP per one chromosome segment, if we apply the formula to this dataset. This causes collinearity and possibly a high variance inflation factor for the estimators, amplified by changes to the effective population size during the selection. While 435 segments suggest that 435 SNP could explain nearly all variation, this is not so as the boundary between segments is fluid.

Meuwissen et al. (2001) have found a small decay in accuracy as the relationship between prediction and training generations decreases in a simulation study. According to the authors this decrease was small enough to maintain the success of breeding schemes after six generations without re-estimation of SNP effects, however, their simulation assumed random mating. Also in a simulation study, Sonesson and Meuwissen (2009) found that re-estimating the genomic effects in every generation can maintain the accuracy of the predictions of breeding values constant. Solberg et al. (2009) also found a decrease in accuracy in further generations. They observed that with a denser panel the decay was smaller, which is probably a consequence of a higher LD between the markers and the simulated QTL. All above mentioned studies did not simulate selection in the data.

Muir (2007) showed that directional selection caused a great decline in accuracy of GEBV, demonstrating that high accuracies in the training generations were not maintained in future generations under selection. This can be a sign that the LD between marker and QTL can be lost across generations under selection, and can result in the changes observed in the present study. Alternatively, the QTL with largest effects are rapidly fixed by selection leaving SNPs with small effects remaining. In a real dataset from layer chicken, Wolc et al. (2011) demonstrated that the decay in accuracy was large enough to require a retrain of the model in every generation. Accurate estimations of genomic breeding value depend on the consistency of LD between markers and QTLs across generations (Calus, 2010), as well as proper SNP effect estimation. The LD is created and maintained by the selection process, among other factors (Lynch and Walsh, 1998). On the other hand, if a change in the allele frequency of two different loci is observed, which can be caused by selection, the LD between them can decrease (Calus, 2010). The results shown in those studies clearly display a loss of genomic prediction accuracy due to the decay of LD. This could also be extended to GWAS, and the negative impact LD decay might have on the accuracy of associations. The variation in the estimates of SNP variance in the present study can be related with those findings, because using values estimated in a different generation would lead to low predictive power if they are not constant.

The small values for SNP effect and percentage of variance explained that were obtained in this study can be related to the findings on Muir et al. (2008). The authors found significant absence of rare alleles in commercial chicken lines. Such findings were related to high inbreeding and consequently to a considerable number of alleles missing, which will reduce the allelic and genetic variability. This narrowed genetic variability can result in weaker associations for the markers, since important alleles could be lost in the process.

The short-term decay in accuracy depends more on the decrease of genomic relationships captured by markers rather than on LD (Habier et al., 2007). Therefore, the accuracy of genomic evaluation is mainly controlled by genomic relationships (Daetwyler et al., 2012; Wientjes et al., 2013). In particular, Daetwyler et al. (2012) found that 86% of the accuracy in genomic selection was retrieved by using SNP from a single chromosome. Subsequently, windows with large effects in Manhattan plots may be an artifact of relationships and not due to LD. The reason why the accuracy does not collapse completely in further generations is that some LD still persists over time, even though selection process and divergence can erode LD. Thus, the observed changes in the SNP effects across the generations in the present study can be a consequence of the changes in the relationship structure across different generations more than decay in LD.

## CONCLUSION

Except for a few regions, the variation explained by the top SNP windows changes over generations. Therefore, even if SNP windows with large variance are detected in a particular data set, their usefulness for genomic selection over many generations is limited. The variance explained by an individual window is not enough to lead selection decisions based on the top regions for the studied traits.

## Conflict of Interest Statement

The authors declare that the research was conducted in the absence of any commercial or financial relationships that could be construed as a potential conflict of interest.
